# Suboptimal osteoporosis care in hospitalized patients: a retrospective analysis of vertebral compression fractures detected on computed tomography

**DOI:** 10.1007/s00296-024-05720-3

**Published:** 2024-09-17

**Authors:** Artem Minalyan, Terrence Li, Kathleena D’Anna, Nasam Alfraji, Lilit Gabrielyan, Christina Downey

**Affiliations:** 1 Division of Rheumatology, Department of Medicine, Loma Linda University Health, Loma Linda, CA USA; 2 Department of Radiology, Loma Linda University Health, Loma Linda, CA USA; 3 Department of Chemistry, University of Redlands, Redlands, CA USA

**Keywords:** Compression fracture, Osteoporosis, Vertebral, Fragility

## Abstract

Vertebral compression fractures (VCFs) are the most common osteoporotic fractures. Only 1/3 of patients with VCFs are clinically diagnosed. In our institution, the Fracture Liaison Service (FLS) was launched in 2017 to improve osteoporosis management for hospitalized patients. (1) To assess osteoporosis awareness among medical providers for emergency department (ED)/hospitalized patients aged 50 or greater; (2) To estimate the rate of FLS consults or referrals to primary care providers (FLS/PCP) by primary teams. A centralized radiology system was used to examine all thoracic and lumbar computed tomography (CT) scans conducted between June 1, 2017 and June 1, 2022. 449 studies were identified with the radiologic impression “compression fracture”. 182 studies were excluded after manual chart review. 267 hospitalizations/ED visits with lumbar and/or thoracic spine CT scans were included. Referrals to FLS (26) or PCP (27) were made in 53 cases (~ 20% of the total). In the ED subgroup (131 hospitalizations), only 17 patients had FLS/PCP referrals. The “compression fracture” was mentioned in 227 (85%) discharge notes (any part), while “osteoporosis” was mentioned in only 74 (28%) hospitalizations. A statistically significant difference was found between the two groups when “osteoporosis” was mentioned in the “assessment and plan” section (p = 0.02). Our data show that the overall osteoporosis care for affected patients is suboptimal. Medical providers often overlook the presence of osteoporosis, leading to a lack of consultation with the FLS of referral to PCPs for further evaluation and treatment.

## Introduction

Osteoporosis is the most common metabolic bone disease in the world. In the United States, half of all women and a quarter of all men will suffer an osteoporotic fracture in their lifetime [1]. With the aging population, the incidence of osteoporosis and subsequent fragility fractures is expected to continue to increase. Of those who suffer a fragility fracture, which is pathognomonic for osteoporosis, only around 11% of patients will receive anti-osteoporotic therapy in the two years that follow the fracture [2]. As dual-energy X-ray absorptiometry (DXA) is underutilized in screening for osteoporosis to implement primary prevention measures, osteoporosis is often diagnosed when the patient has their first fragility fracture. In the United States, vertebral compression fractures (VCFs) are the most common osteoporotic fractures, with an estimated 700,000 occurring per year [3]. To diagnose a patient with osteoporosis after a radiographically identified compression fracture, there are two steps that must occur. First, the radiologist must recognize and report the vertebral compression fracture. Second, the person reading the report must recognize that a VCF is tantamount to a diagnosis of osteoporosis. Only after the diagnosis is made can the patient be offered anti-osteoporotic therapy.

VCFs may be seen as incidental findings on studies ordered for other purposes, however radiologists may not recognize or report these fractures [4–6]. Studies show that approximately half the time, radiologists fail to recognize osteoporotic vertebral fractures in imaging studies [1]. As these fractures are unreported, a care gap exists resulting in inadequate diagnosis of osteoporosis. When VCFs are reported on imaging studies, only one-third of patients with compression fractures are clinically diagnosed with osteoporosis, which represents another gap in osteoporosis recognition and subsequent treatment initiation [1].

There is a known care gap that exists between the presence of a fragility fracture and subsequent treatment for osteoporosis, both globally and at our institution. To improve osteoporosis recognition and treatment at our hospital, a Fracture Liaison Service (FLS) was instituted in 2017 which improved treatment rates from 10% prior to implementation to nearly 20% post implementation [7]. In this system, all patients with a fragility fracture over the age of 50 should receive an FLS consultation to coordinate care in the outpatient setting for osteoporosis treatment. Currently, there is no mechanism to generate an FLS consultation from radiology reports of VCFs.

However, not all imaging modalities are created equal when it comes to VCF recognition. Determining if a VCF is due to osteoporosis, rather than trauma or malignancy, x-ray is not as sensitive as advanced imaging techniques such as computed tomography (CT), magnetic resonance imaging (MRI), and others [2]. Even on these more sensitive imaging modalities, VCFs are underreported on imaging. However, we believe that even in cases of reported VCFs, the awareness of medical teams taking care of affected patients is suboptimal, leading to inadequate and delayed osteoporosis management.

This study aims to determine the outcomes in patients who have had a CT scan in the emergency room or inpatient setting that showed a reported VCF. Our study recorded if the primary team reported osteoporosis on the discharge summary and if the primary team referred the patient to the FLS while hospitalized or to their primary care doctor for osteoporosis treatment upon discharge.

## Materials and methods

### Patient selection

A centralized radiology report archival system (“Montage Analytics”) was used to examine all radiologic reports of CT scans of the lumbar and/or thoracic spine within the period from 06/01/2017 to 06/06/2022. The key phrase “compression fracture” in the impression section of radiology reports was searched. Included patients were aged 50 years and older who had CT scans of the thoracic and/or lumbar spine in the emergency department (ED) or while hospitalized. Exclusion criteria were (1) patients with suspected non-osteoporotic fracture (malignancy, infection, post-traumatic), (2) those who did not have compression fractures after manual review of impressions with the key phrase “compression fracture”, (3) patients who were transferred to other hospitals, and (4) those who expired during hospitalization or were discharged to hospice. Additionally, we excluded CT duplicate studies (patients who had concurrent thoracic and lumbar spine CT scans during the same hospitalization).

We compared patients who either had FLS consultations or were referred to their PCP (Primary Care Physician) with those who did not have FLS/PCP involvement for osteoporosis management. The electronic medical record (EMR) software, “Epic Systems,” was used to review patient records. The following variables were included in the data analysis: patient age, gender, primary language, race, dates of admission and discharge, date of CT study, body-mass index (BMI), presence of prior fractures (as mentioned in the CT reports of interest with reference to prior comparison studies), number of new fractures on CT reports, primary team (emergency medicine (EM), internal medicine (IM), orthopedic surgery, etc.), reason for hospitalization (fall ± syncope, back pain, other pain, other causes), patient disposition (home, skilled nursing facility, acute rehab), compression fracture mentioned in discharge summary ± “assessment and plan” section of discharge summary, osteoporosis mentioned in discharge summary ± “assessment and plan” section of discharge summary, and referral to PCP or consulting FLS during hospital stay.

The study was approved by the Loma Linda Institutional Review Board (IRB #5,230,262, approval date: July 19, 2023). A waiver of informed consent was granted due to the retrospective nature of the study.

### Statistical analysis

IBM SPSS software (version 29) was used for statistical analysis. Chi-tests were performed for all categorical (nominal) variables, including sex, language, race, reason for hospitalization, etc. A Welch t-test was used for quantitative variables, including age, length of hospitalization, and BMI. A 95 percent confidence interval (CI) and a p-value of < 0.05 were used to determine statistical significance.

## Results

**Fig. 1:**
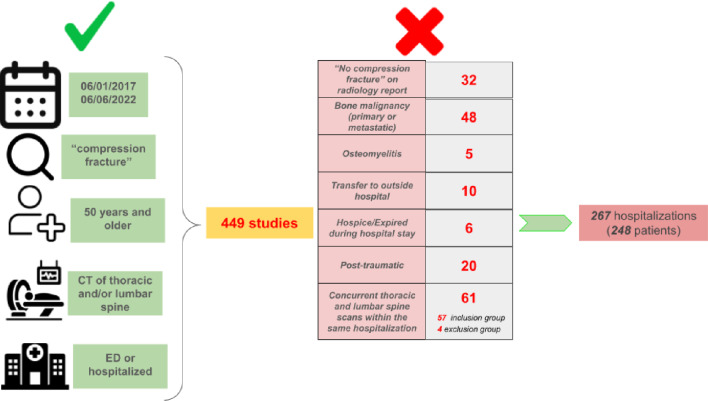
Patient selection for the study with listed inclusion and exclusion criteria. *CT* computed tomography, *ED* emergency department

Overall, 449 studies were reviewed using the inclusion criteria. After applying the exclusion criteria, 267 hospitalizations (involving 248 unique patients) were included for further data collection and analysis (Fig. 1).

Out of 248 patients, multiple (> 1) hospitalizations (or ED visits) were found in 13 patients. Among these, 9 patients had 2 encounters, 2 patients had 3 encounters, and another 2 patients had 4 encounters. Only 3 patients (out of 13) had FLS consulted during hospitalization (or were referred to PCP): 2 patients had 4 hospitalizations, and 1 patient had 3 hospitalizations.

Overall, we identified only 53 (~ 19.9%) hospitalizations (out of 267) in which patients were either referred to PCP (27) or had FLS (26) consulted during the hospitalization. Most hospitalizations involved female patients (64%). In 13.4% of male hospitalizations (13 out of 97) and 23.5% of female hospitalizations (40 out of 170), referrals to PCP or FLS consultations were made. There was no statistically significant difference in the length of hospitalization, BMI, primary language, race, or reason for hospitalization between FLS/PCP and no FLS/PCP groups. However, there was a statistically significant difference in the primary teams between the two groups (p = 0.04). The primary team was responsible for the discharge summary of the affected patient. Notably, 49% of hospital visits did not result in hospitalizations (ED visits only). Only around 13% of ED discharge notes had FLS consults or PCP referrals. In contrast, 36.4% of patients on the orthopedic surgery team had FLS/PCP involvement.

**Fig. 2:**
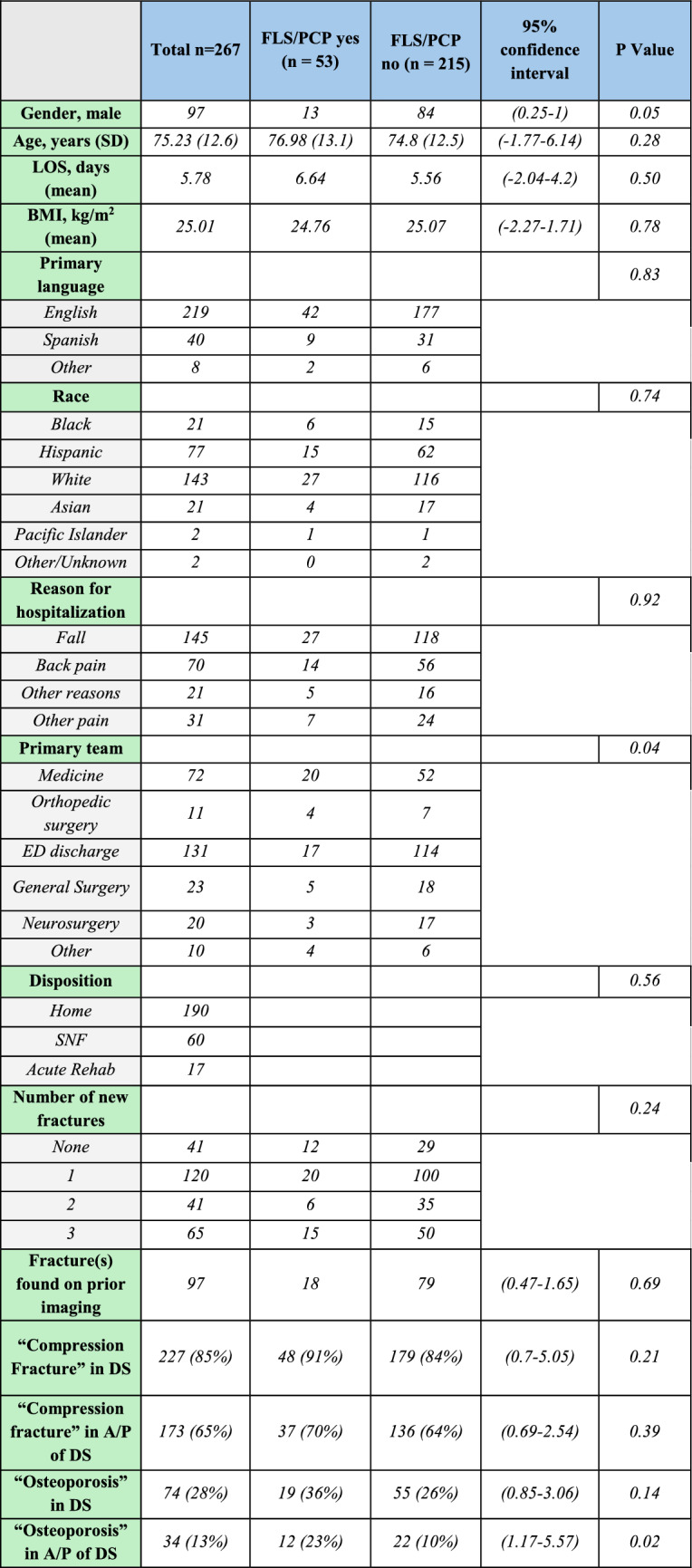
Characteristics of patients with vertebral compression fractures during the study period. *LOS* length of stay, *DS* discharge summary, *A/P* assessment and plan, *FLS* fracture liaison service, *PCP* primary care provider, *ED* emergency department, *SNF* skilled nursing facility, *BMI* body-mass index, *SD* standard deviation

We did not find any difference between the two grops when comparing the disposition of patients, the mention of prior fractures on CT reports, or the number of new fractures. Notably, the wording in the discharge summary that included “compression fracture” anywhere in the note, including the “assessment and plan” section, did not show a statistically significant difference when comparing the two groups. However, if the term “osteoporosis” was mentioned in the “assessment and plan” section of the discharge summary, there was a statistically significant difference involving FLS/PCP (23% vs. 10%, p = 0.02). It is worth mentioning that “compression fracture” was included in 85% of discharge summaries, while the term “osteoporosis” was found only in 28% of discharge summaries (Fig. 2).

**Fig. 3:**
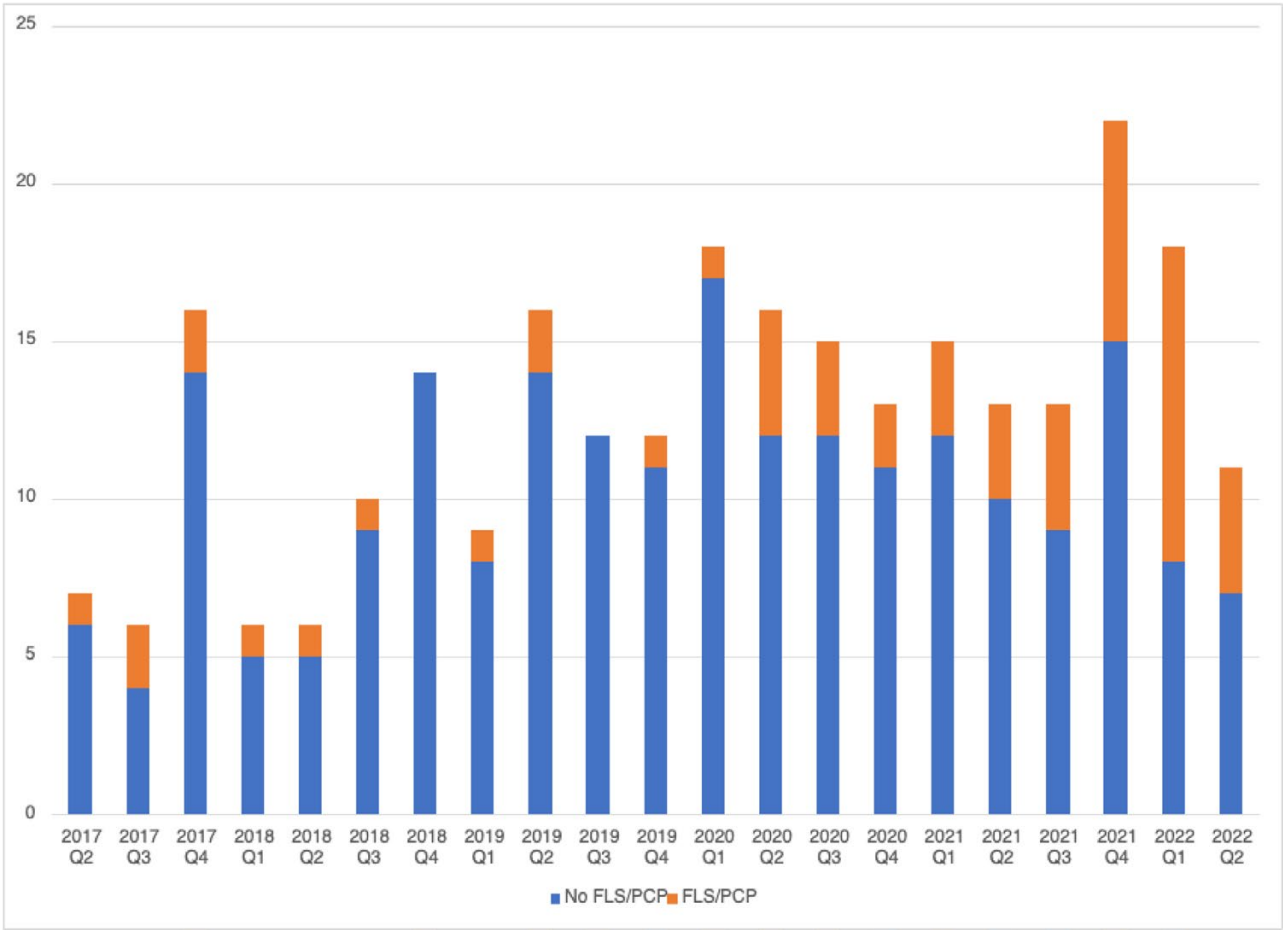
The trend of hospitalizations with vertebral compressions fractures detected on CT spine and FLS consultations/PCP referrals during the study period. (horizontal axis: time in quarterly periods; vertical axis: number of hospitalizations) *FLS* fracture liaison service, *PCP* primary care physician

An overall trend of hospitalizations with compression fractures in the thoracic and lumbar spine, and the rate of FLS/PCP referrals during the study period, was also assessed (Fig. 3). It shows that the overall rate of FLS/PCP referrals was lower initially when the FLS was launched in our institution and increased over time. During the peak of COVID-19 hospitalizations (2020: Q2, Q3; 2021: Q1), we identified more FLS/PCP referrals (relative to all VCF hospitalizations during that period) compared to some of the pre-COVID-19 periods. For instance, during two of the quarters (2018: Q4; 2019: Q3), no FLS/PCP referrals were made for hospitalized patients with VCFs.

## Discussion

It is estimated that an osteoporotic fracture occurs every 3 s worldwide [8]. According to the World Health Organization (WHO), the global population aged 60 years and above will reach 2.1 billion, including 426 million people aged 80 years and above, by 2050 [9]. The term “osteoporosis” (from Ancient Greek “osteon” (bone) and “porosis” (pore, petrification)) was first introduced in the beginning of the nineteenth century in France. The word was subsequently adopted in the English medical literature in the twentieth century [10]. Not surprisingly, our understanding of the disease has evolved over the past two centuries. The diagnosis of osteoporosis in men aged 50 years and above and postmenopausal women does not require performing bone mineral density (BMD) if there is a history of low trauma (fragility) fracture.

The most common mechanism of a fragility fracture is a fall from a standing height or less [11]. Fragility fractures of the hip and spine have the most profound impact on the health of the affected patients [12]. It is estimated that VCFs are the most common osteoporotic fractures among both men and women. It is suggested that up to 65–75% of VCFs may be clinically “silent” [13, 14]. In addition, many VCFs are underreported by radiologists. Li et al*.* reported that 66.8% of patients with VCFs on lateral chest x-ray were undiagnosed in their initial radiology reports [5]. Black et al*.* showed an associated fivefold risk of sustaining a subsequent fracture in women aged 65 years and older after the initial VCF [15]. Furthermore, the presence of VCFs is associated with an increase in mortality that persists beyond one year, resulting in a 5 year survival rate of 56.5% compared to the expected 69.9% [16]. It has been reported that patients with rheumatic and musculoskeletal diseases are at increased risk of osteoporosis and related fractures compared to individuals without those conditions [17, 18]. Long-term rheumatoid arthritis is now considered a risk factor for refracture in patients with known fragility fractures [19]. Fragility fractures are more common in patients with lupus who have hematologic involvement (thrombocytopenia, hemolytic anemia) [20]. In addition, gout has been found to be an independent risk factor for the development of thoracic vertebral fractures [21].

The diagnostic delay due to underreporting of VCFs is well known. However, the impact of the awareness of the osteoporotic fracture by non-radiologists in reported VCFs in hospitalized patients or those managed in the ED has not been well described. We hypothesized that VCFs found on advanced radiology reports (CT lumbar and/or thoracic spine) during a hospital stay do not receive adequate recognition. This can lead to significant delay in the diagnosis of osteoporosis with subsequent additional vertebral or non-vertebral fractures, including hip fractures.

Our study showed that less than 20% of hospitalizations in which VCFs were reported on CT resulted in consulting FLS or referring the patient to PCP for subsequent management of osteoporosis. Even among patients with recurrent hospitalizations (13 patients), the utilization of FLS/PCP was surprisingly low. In fact, only 3 patients (out of 13) had FLS consulted (or referred to PCP) during their hospitalization. Further chart review showed that 2 of those patients had 3 hospitalizations and 1 patient had 4 hospitalizations during the study period with CT thoracic and/or lumbar spine performed during each hospitalization. The lowest percentage of FLS/PCP utilization for VCFs was by the EM teams (13%). The highest involvement of FLS (or referral to PCP) was observed in patients on the orthopedic surgery teams. However, even in the latter group, the percentage was incredibly low (36.4%).

The reason for hospitalization, the presence of prior VCFs, and the number of new VCFs did not provide any statistically significant difference in utilizing FLS/PCP in affected patients. We also observed poor recognition among healthcare providers that a vertebral compression fracture is diagnostic of osteoporosis. The term “compression fracture” was used in 85% of discharge summaries of hospitalizations. However, the term “osteoporosis” was mentioned only in 28% of all hospitalizations with VCFs. Notably, when the term “osteoporosis” was used in the “assessment and plan” section of discharge summaries, there was a statistically significant difference in the utilization of FLS/PCP during those hospitalizations (p = 0.02).

In our study, we meticulously reviewed the charts of patients with reported VCFs that were hospitalized or treated only in the ED. We manually reviewed all complete radiology reports (in addition to impressions) to identify any possible non-osteoporotic compression fractures that were excluded from the study. We particularly selected the study duration to reflect the launch of FLS service in our institution (beginning of 2017) and peak of the COVID-19 pandemic and subsequent decrease of COVID-19-related hospitalizations.

Despite being a single-center study, we did not have access to all outpatient records of the affected patients either prior to or after hospitalizations. That is because some patients may have received outpatient osteoporosis management by their PCPs not affiliated with our institution and thus capturing these patients was difficult. In addition, some of the initial low utilization of FLS could be explained by poor awareness of medical providers in our institution of FLS when it was first launched in 2017. (Fig. 3). Our data clearly show a concerning trend where, despite the mention of “compression fracture” in the impression of CT reports, the overall osteoporosis care for affected patients was suboptimal. It could be partly due to the lack of recognition of compression fracture as osteoporosis in affected patients.

With the advances of machine learning in medicine as well as recognition of diagnostic and therapeutic delays resulting from human errors, we believe that implementing certain alert protocols within the electronic medical software could potentially improve care for patients with VCFs. Some of the changes could include the use of certain phrases by radiologists in their reports. For instance, they could state that “further evaluation for possible osteoporotic nature of the compression fracture is needed.” Another option would be flagging patients with VCFs by the radiology team in the electronic medical software. That can be followed by the subsequent review by the FLS team to decrease the dependence on timely recognition of compression fractures and osteoporosis awareness by primary teams during hospitalizations.

## Data Availability

Raw data can be shared if requested.
